# Degree of urbanization and the prevalence of hypertension and diabetes mellitus in South Korea (Insight from Gyeonggi Province): A cross-sectional analysis with temporal trends, 2008–2023

**DOI:** 10.1186/s12889-026-28174-7

**Published:** 2026-06-19

**Authors:** Sang-Suk Choi, Jin Jung, Ji-Hyun Kim, Soo-Yeon Jung, Ki-Dong Yoo, Donggyu Moon, Su-Nam Lee, Won-Young Jang, Ji-Hoon Jung, Sung-Ho Her

**Affiliations:** 1 Division of Cardiology, Department of Internal Medicine, St. Vincent’s Hospital, The Catholic University of Korea, 93, Jungbu-daero, Paldal-gu, Seoul, Suwon, Gyeonggi-do 16247 Republic of Korea; 2 Center for National Toxicology Artificial Intelligence and Data, Korea Institute of Toxicology, Daejeon, Republic of Korea

**Keywords:** Hypertension, Diabetes Mellitus, Degree of Urbanization, Health Status Disparities, Cross-Sectional Studies, South Korea

## Abstract

**Background:**

Despite South Korea’s achievement of Universal Health Coverage (UHC), regional health inequities persist as a significant public health challenge. Traditional urban-rural classifications often fail to capture the nuanced spatial dynamics of modern settlement patterns. This study aimed to evaluate the prevalence of hypertension (HTN) and diabetes mellitus (DM) across the urbanization gradient—Cities, Urbanized, and Rural areas—using the UN-recommended Degree of Urbanization (DegUrba) classification.

**Methods:**

We analyzed survey-weighted data from the Korean Community Health Survey (KCHS), restricting cross-sectional analyses to adults aged ≥ 18 years residing in Gyeonggi-do in 2020 (*n* = 41,983), classified into three DegUrba categories (Urban, Urbanized, Rural) using 1-km² population grids. Baseline characteristics were compared using survey-weighted linear regression and the Rao–Scott chi-square test. Three survey-weighted logistic regression models were constructed, with the fully adjusted model (Model 3: age, sex, education, marital status, smoking, and alcohol consumption) as the primary model. A sensitivity analysis excluded municipalities whose DegUrba classification changed during the preceding 10-year period. Age-standardized prevalence estimates from 2008 to 2023 were calculated by direct standardization to the 2020 study population, and temporal trends were assessed using general linear models.

**Results:**

In 2020, the weighted prevalence of hypertension and diabetes mellitus was highest in Rural areas (29.4% and 13.4%) and similar between Urban (19.4% and 8.3%) and Urbanized (20.1% and 8.6%) areas (overall *P* < 0.001 for both). In the fully adjusted Model 3 with Urban as reference, the rural disadvantage was attenuated and only borderline significant (HTN: OR 1.09, 95% CI 0.99–1.21, *P* = 0.077; DM: OR 1.11, 95% CI 0.97–1.27, *P* = 0.143), whereas with Urbanized as reference the association was statistically significant for hypertension (OR 1.14, 95% CI 1.02–1.28, *P* = 0.026). The Urban–Urbanized comparison was not significant across all models. The sensitivity analysis restricted to municipalities with stable DegUrba classification yielded stronger and more consistent rural effects (HTN Model 3 vs. Urbanized: OR 1.16, *P* = 0.014; DM: OR 1.18, *P* = 0.049). From 2008 to 2023, age-standardized prevalence increased significantly for both conditions (year *P* = 0.002 for HTN, *P* < 0.001 for DM); Urban and Urbanized prevalences remained statistically indistinguishable (*P* > 0.999), whereas Rural prevalence was higher than both (all *P* < 0.005).

**Conclusion:**

The cross-sectional urban–urbanized similarity and persistently higher rural prevalence — particularly in geographically stable rural municipalities — indicate that the conventional urban–rural binary still captures most of the cardiometabolic disparity, but that compositional differences (age, education, marital status) account for a substantial portion of the raw rural penalty. Under universal health coverage, residual rural disadvantage in cardiometabolic disease remains a public-health priority, especially as the rural–urban gap continues to widen over time.

**Supplementary Information:**

The online version contains supplementary material available at https://doi.org/10.1186/s12889-026-28174-7.

## Introduction

Hypertension (HTN) and diabetes mellitus (DM) represent the leading global risk factors for cardiovascular disease (CVD), contributing significantly to premature mortality and escalating healthcare costs [1, 2]. Globally, the epidemiological landscape of these cardiometabolic diseases has shifted; while historically associated with high-income urban centers, recent large-scale evidence—notably from the PURE study—indicates that the burden is rapidly increasing in rural areas of middle-income and high-income nations [3, 4]. In South Korea, the rapid demographic shift toward an ultra-aged society has intensified the socioeconomic burden of these diseases, making effective community-level management a critical priority for national healthcare sustainability [5, 6].

South Korea’s healthcare system is characterized by a robust Universal Health Coverage (UHC) framework, which, in principle, provides equitable medical access to all citizens [7]. However, evidence from various developed nations suggests that geographical location remains a significant barrier to health equity even under unified insurance systems [8, 9]. Most existing literature in Korea has relied on administrative boundaries (e.g., Dong vs. Eup/Myeon) to define regionality. This binary classification is increasingly criticized for its inability to reflect actual population density and functional settlement patterns, often masking the heterogeneity of rapidly developing “semi-dense” areas. Similar limitations have been observed in other regions; for instance, studies in China and Brazil have demonstrated that traditional administrative labels often obscure the health profiles of “peri-urban” zones where urbanization is actively transitioning [10, 11].

To address this gap, international organizations including the United Nations (UN) and the European Commission developed the “Degree of Urbanization” (DegUrba) framework [12]. Unlike traditional administrative divisions, DegUrba utilizes high-resolution population grids (1 km x 1 km) to categorize regions based on actual settlement density and contiguity. By moving beyond a simple urban-rural dichotomy and adopting the concept of an “urbanization gradient,” this framework allows for a more granular and sophisticated analysis of regional disparities. By applying this standardized metric to Gyeonggi-do, we can more precisely evaluate whether the “urban-rural gap” is driven by residential density or underlying social determinants, thereby identifying subtle health variations that were previously obscured. Gyeonggi-do was selected as the target region because it represents the most populous and rapidly developing area in South Korea. According to the 2020 Population and Housing Census conducted by Statistics Korea, Gyeonggi-do houses approximately 13.4 million residents, accounting for 25.9% of the entire national population (51.8 million), despite occupying only about 10.1% of the country’s land area. This region features a unique mix of highly developed urban centers, large-scale new towns, and rural communities, making it an ideal setting to investigate the health impacts of rapid urbanization.

Our study had three sequential, descriptive objectives. First, we sought to overcome the limitations of the conventional administrative urban–rural dichotomy by applying the standardized UN-DegUrba framework to a single, well-defined province (Gyeonggi-do). Second, we aimed to quantify the spatial heterogeneity of cardiometabolic disease burden—specifically the prevalence of hypertension (HTN) and diabetes mellitus (DM)—across the resulting urbanization gradient (Cities, Urbanized, and Rural areas). Third, by additionally evaluating temporal trends in age-standardized prevalence from 2008 to 2023, we aimed to determine whether the cross-sectional disparity is stable, widening, or narrowing over the recent 16-year period. We hypothesized that, despite the country’s universal health coverage, prevalence of HTN and DM would be highest in Rural areas, and that part of this rural excess would be explained by compositional differences in age and socioeconomic indicators.

## Methods

### Data source and sampling design

This study used data from the Korean Community Health Survey (KCHS), a nationwide, community-based cross-sectional survey conducted annually since 2008 by the Korea Disease Control and Prevention Agency (KDCA) [13]. The KCHS employs a complex multistage stratified cluster sampling design with probability proportion-to-size (PPS) systematic sampling within Gyeonggi-do. All analyses accounted for the survey design using the stratification variable, primary sampling unit, and individual sampling weights. Data collection was performed by trained interviewers using a Computer-Assisted Personal Interviewing (CAPI) system. Cross-sectional analyses were restricted to adults aged ≥ 18 years residing in Gyeonggi-do in 2020 (*n* = 41,983). For temporal trend analyses, all KCHS waves from 2008 to 2023 were pooled.

The annual Gyeonggi-do sample size in KCHS ranged approximately from 18,000 to 41,000 across the 2008–2023 waves, with sampling weights adjusted yearly to maintain representativeness of the Gyeonggi-do adult population. Sampling weights and design variables were applied separately within each year, and age standardization to the 2020 study population was used to ensure comparability across waves.

### Regional classification: The DegUrba framework

Regional classification was based on the DegUrba methodology [12]. Cities (Urban Centers) are areas where at least 50% of the population resides in high-density clusters (density > 1,500 inhabitants/ 1 km²; minimum population 50,000). Urbanized areas are where less than 50% live in an urban center but at least 50% reside in an urban cluster (density > 300 inhabitants/1km²; minimum population 5,000). Rural Areas encompass regions where most of the population lives in rural grid cells outside both urban centers and urban clusters. In accordance with this classification, we categorized the regions within Gyeonggi-do into these three groups—Cities, Urbanized and Rural Areas—for our analysis in this study.

Regional category was assigned based on the participant’s registered residence at the time of the survey, as captured by the standardized KCHS questionnaire; workplace location and duration of residence were not used in the assignment. In the primary analysis, all participants were classified by their 2020 DegUrba category. To address potential misclassification due to the dynamic nature of urbanization in Gyeonggi-do, a sensitivity analysis was performed in which municipalities whose DegUrba classification changed during the preceding 10-year period were excluded. The resulting geographic distribution of the three DegUrba categories across the municipalities of Gyeonggi-do is shown in Supplementary Fig. 1.

### Definition of outcomes and covariates

The primary outcomes were the point prevalence of HTN and DM as of 2020, ascertained exclusively via self-reported physician diagnosis using the standardized KCHS questionnaire; specifically, participants were asked whether they had ever been told by a physician that they had hypertension or diabetes. Direct clinical measurements (blood pressure, fasting plasma glucose, or HbA1c) are not collected as part of KCHS. Pregnancy-related conditions—gestational hypertension and gestational diabetes mellitus—were neither queried as separate items in the KCHS questionnaire nor classified as hypertension or diabetes in the present analysis.

Covariates included age, sex, and socioeconomic indicators (education and marital status); sex was defined as sex assigned at birth as recorded by the survey instrument. Lifestyle variables (smoking status and alcohol consumption) were dichotomized into current smokers versus non-smokers, and current drinkers versus non-drinkers, respectively, and applied consistently throughout the manuscript and tables. Current smokers were defined as individuals who reported smoking ≥ 100 cigarettes in their lifetime and currently smoking; all others were classified as non-smokers, consistent with the KCHS standardized definition. Current drinkers were defined as participants who reported any alcohol consumption in the past 12 months.

### Statistical analysis

All statistical analyses were performed using SAS software version 9.4 (SAS Institute Inc., Cary, NC, USA). Continuous variables are presented as weighted means with standard errors (SE), and categorical variables are presented as unweighted frequencies with weighted percentages. Baseline characteristics across the three DegUrba categories were compared using survey-weighted linear regression for continuous variables and the Rao–Scott chi-square test for categorical variables.

The associations between DegUrba and HTN/DM were assessed using survey-weighted logistic regression. Crude and adjusted odds ratios (ORs) with 95% confidence intervals (CIs) were estimated. Three multivariable models were constructed: Model 1 was adjusted for age and sex; Model 2 was adjusted for age, education level, marital status, and alcohol consumption; and Model 3 was additionally adjusted for sex and smoking status. Age was included as a continuous variable in all regression models. Model 3 was considered the primary model. To facilitate interpretation of pairwise comparisons, results were presented separately with Urban and with Urbanized as reference categories. As a sensitivity analysis, municipalities whose DegUrba classification changed during the preceding 10-year period were excluded, and the primary analyses were repeated.

For the temporal trend analysis from 2008 to 2023, age-standardized prevalence estimates for HTN and DM were calculated using the direct standardization method, with the age distribution of the 2020 study population as the standard population. Temporal trends were evaluated using a general linear model including year, DegUrba category, and their interaction term. P-values are reported for the linear effect of year, the overall effect of DegUrba category, and the year × DegUrba interaction. Pairwise comparisons among the three DegUrba categories were adjusted using the Bonferroni correction. A two-sided P-value < 0.05 was considered statistically significant.

### Quality control

Standardized quality-control procedures of the KCHS were applied throughout data collection and processing. Field interviewers received standardized pre-survey training and annual re-certification, and Computer-Assisted Personal Interviewing (CAPI) was used to enforce real-time range checks, skip patterns, and consistency rules at the point of data entry. Completed questionnaires underwent a two-stage review—first on-site by regional supervisors and subsequently centrally by the Korea Disease Control and Prevention Agency (KDCA)—and any inconsistencies identified at either stage were resolved through telephone call-back verification with the respondent. The KCHS is registered as an Approved National Statistic by Statistics Korea (Approval No. 117075), which mandates ongoing methodological audit and external review. For the present analysis, all variables of interest were further screened for out-of-range and logically inconsistent values prior to applying complex sampling weights; no records required exclusion based on these checks.

## Results

### Baseline characteristics

Among the 41,983 adults aged ≥ 18 years residing in Gyeonggi-do who participated in the 2020 wave of the Korean Community Health Survey, all eligible respondents were retained for analysis; no participants were excluded due to missing data on the key covariates of interest (age, sex, education, marital status, smoking, alcohol consumption, hypertension, and diabetes mellitus status) and 30,178 (71.9%), 8,189 (19.5%), and 3,616 (8.6%) were classified as residing in Urban, Urbanized, and Rural areas, respectively (Table 1, Supplementary Fig. 1). Rural residents were on average older than Urban or Urbanized residents (mean age 53.5 ± 0.4 vs. 46.9 ± 0.1 and 48.0 ± 0.3 years, *P* < 0.001) and were more likely to have completed only middle school or less (29.8% vs. 15.6% and 16.1%, *P* < 0.001) and to be non-drinkers (38.2% vs. 30.5% and 32.2%, *P* < 0.001). Sex distribution and smoking status did not differ significantly across the three categories. The weighted prevalence of hypertension was 19.4%, 20.1%, and 29.4% in Urban, Urbanized, and Rural areas, respectively, and the corresponding prevalence of diabetes mellitus was 8.3%, 8.6%, and 13.4% (both *P* < 0.001).

**Table 1:** Baseline characteristics of the study population by degree of urbanization (DegUrba) in 2020

Variable	Category	Urban (*n* = 30,178)	Urbanized (*n* = 8,189)	Rural (*n* = 3,616)	*P*-value
		*n* (%)	*n* (%)	*n* (%)	
Hypertension		6,356 (19.4)	1,884 (20.1)	1,248 (29.4)	< 0.001
Diabetes mellitus		2,688 (8.3)	843 (8.6)	556 (13.4)	< 0.001
Age, mean ± SE (years)		46.9 ± 0.1	48.0 ± 0.3	53.5 ± 0.4	< 0.001
Age group (years)					< 0.001
	18–29	4,989 (18.1)	1,174 (16.9)	358 (13.0)	
	30–39	4,534 (17.9)	1,083 (16.1)	322 (11.3)	
	40–49	6,099 (20.8)	1,670 (21.8)	414 (15.7)	
	50–59	6,320 (20.3)	1,696 (19.7)	685 (20.6)	
	60–69	4,533 (12.9)	1,325 (13.6)	826 (17.9)	
	70–79	2,568 (7.0)	857 (8.4)	660 (14.3)	
	≥ 80	1,135 (3.0)	384 (3.6)	351 (7.3)	
Sex					0.685
	Male	13,870 (50.1)	3,819 (50.0)	1,714 (50.8)	
	Female	16,308 (49.9)	4,370 (50.0)	1,902 (49.2)	
Education level					< 0.001
	Middle school or less	5,586 (15.6)	1,805 (16.1)	1,376 (29.8)	
	High school	9,318 (30.7)	2,566 (29.5)	1,083 (31.8)	
	College or more	15,161 (53.6)	3,796 (54.4)	1,155 (38.4)	
Marital status					< 0.001
	Single	7,174 (25.9)	1,648 (23.0)	533 (18.6)	
	Married	18,561 (61.2)	5,164 (62.7)	2,317 (63.1)	
	Divorced/Widowed/Separated	4,414 (13.0)	1,369 (14.3)	766 (18.3)	
Smoking status					0.438
	Non-smoker	24,942 (81.1)	6,674 (80.8)	2,980 (79.7)	
	Current smoker	5,236 (18.9)	1,515 (19.2)	636 (20.3)	
Alcohol consumption					< 0.001
	Non-drinker	9,943 (30.5)	2,929 (32.2)	1,559 (38.2)	
	Drinker	20,235 (69.5)	5,260 (67.8)	2,057 (61.8)	

Continuous variables are presented as weighted mean ± standard error (SE); categorical variables are presented as unweighted frequencies with weighted percentages. P-values were calculated using survey-weighted linear regression for continuous variables and the Rao–Scott chi-square test for categorical variables. Age was categorized into 10-year groups, with the youngest group defined as 18–29 years because the Korean Community Health Survey includes adults aged ≥ 18 years

*DegUrba* Degree of Urbanization, *SE* Standard error

### Survey-weighted logistic regression

**Fig. 1:**
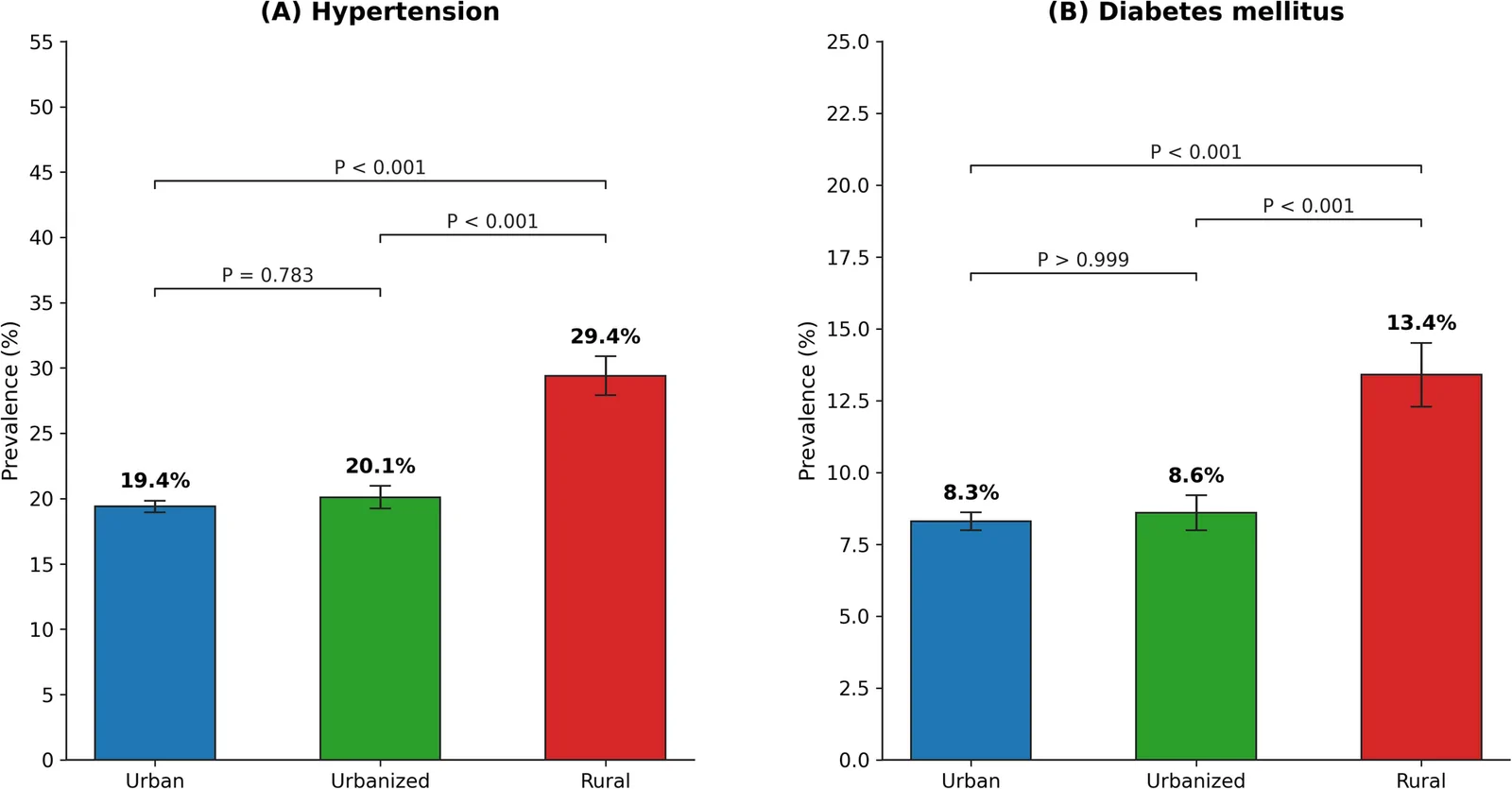
Prevalence of (**A**) hypertension and (**B**) diabetes mellitus by degree of urbanization (DegUrba) in 2020. Bars represent survey-weighted prevalence in 2020. Error bars indicate 95% confidence intervals of the weighted proportion. Pairwise P-values were obtained from survey-weighted univariable logistic regression and were Bonferroni-corrected for three comparisons. DegUrba, Degree of Urbanization

In survey-weighted univariable logistic regression with Urban as reference, Urbanized residence was not significantly associated with either condition (HTN: OR 1.04, 95% CI 0.97–1.12, *P* = 0.261; DM: OR 1.04, 95% CI 0.95–1.15, *P* = 0.396), whereas Rural residence was strongly associated with both outcomes (HTN: OR 1.73, 95% CI 1.58–1.88, *P* < 0.001; DM: OR 1.71, 95% CI 1.50–1.95, *P* < 0.001) (Table 2; Fig. 1).

**Table 2:** Survey-weighted logistic regression analyses for the association between the degree of urbanization and hypertension or diabetes mellitus

Outcome	DegUrba	Univariable		Model 1		Model 2		Model 3	
		OR (95% CI)	*P*	OR (95% CI)	*P*	OR (95% CI)	*P*	OR (95% CI)	*P*
Hypertension	Urban	*Reference*		*Reference*		*Reference*		*Reference*	
	Urbanized	1.04 (0.97–1.12)	0.261	0.95 (0.88–1.03)	0.195	0.96 (0.89–1.04)	0.289	0.96 (0.89–1.04)	0.306
	Rural	1.73 (1.58–1.88)	< 0.001	1.10 (1.00–1.22)	0.055	1.10 (1.00–1.22)	0.055	1.09 (0.99–1.21)	0.077
Diabetes mellitus	Urban	*Reference*		*Reference*		*Reference*		*Reference*	
	Urbanized	1.04 (0.95–1.15)	0.396	0.97 (0.87–1.07)	0.516	0.98 (0.88–1.09)	0.689	0.98 (0.88–1.09)	0.723
	Rural	1.71 (1.50–1.95)	< 0.001	1.13 (0.98–1.29)	0.092	1.12 (0.98–1.28)	0.103	1.11 (0.97–1.27)	0.143
Hypertension	Urban	0.96 (0.89–1.03)	0.261	1.05 (0.97–1.14)	0.195	1.04 (0.97–1.13)	0.289	1.04 (0.96–1.13)	0.306
	Urbanized	*Reference*		*Reference*		*Reference*		*Reference*	
	Rural	1.66 (1.50–1.84)	< 0.001	1.16 (1.03–1.30)	0.011	1.15 (1.03–1.29)	0.017	1.14 (1.02–1.28)	0.026
Diabetes mellitus	Urban	0.96 (0.87–1.06)	0.396	1.04 (0.93–1.15)	0.516	1.02 (0.92–1.13)	0.689	1.02 (0.92–1.13)	0.723
	Urbanized	*Reference*		*Reference*		*Reference*		*Reference*	
	Rural	1.64 (1.41–1.90)	< 0.001	1.17 (0.99–1.37)	0.058	1.14 (0.98–1.34)	0.092	1.13 (0.96–1.32)	0.131

Model 1 was adjusted for age and sex. Model 2 was adjusted for age, education level, marital status, and alcohol consumption. Model 3 was additionally adjusted for sex and smoking status (age, sex, education, marital status, smoking, and alcohol consumption). Age was modeled as a continuous variable in all regression models. Model 3 is the primary model. Two reference categories are presented: the upper section uses Urban as the reference category, and the lower section uses Urbanized as the reference category

*OR* Odds ratio, *CI* Confidence interval

After progressive multivariable adjustment, the Urban–Urbanized similarity was preserved across all models, and the rural excess attenuated substantially. In the fully adjusted Model 3 with Urban as reference, the rural association became borderline for HTN (OR 1.09, 95% CI 0.99–1.21, *P* = 0.077) and non-significant for DM (OR 1.11, 95% CI 0.97–1.27, *P* = 0.143). When Urbanized was used as the reference category, the rural disadvantage remained statistically significant in Model 3 for HTN (OR 1.14, 95% CI 1.02–1.28, *P* = 0.026) but not for DM (OR 1.13, 95% CI 0.96–1.32, *P* = 0.131). These findings indicate that compositional differences in age, education, marital status, and lifestyle factors account for the majority of the crude rural penalty, with a small residual contextual association remaining for hypertension.

### Sensitivity analysis

When the analysis was restricted to municipalities with stable DegUrba classification over the preceding 10-year period, the rural disadvantage strengthened and was statistically significant in the fully adjusted models (Supplementary Table 1). With Urban as reference, Model 3 ORs for Rural residence were 1.10 (95% CI 1.00–1.22, *P* = 0.062) for HTN and 1.16 (95% CI 1.00–1.33, *P* = 0.044) for DM. With Urbanized as reference, the Model 3 ORs were 1.16 (95% CI 1.03–1.30, *P* = 0.014) for HTN and 1.18 (95% CI 1.00–1.39, *P* = 0.049) for DM. Urban–Urbanized comparisons remained non-significant, consistent with the primary analysis.

### Temporal trends (2008–2023)

**Fig. 2:**
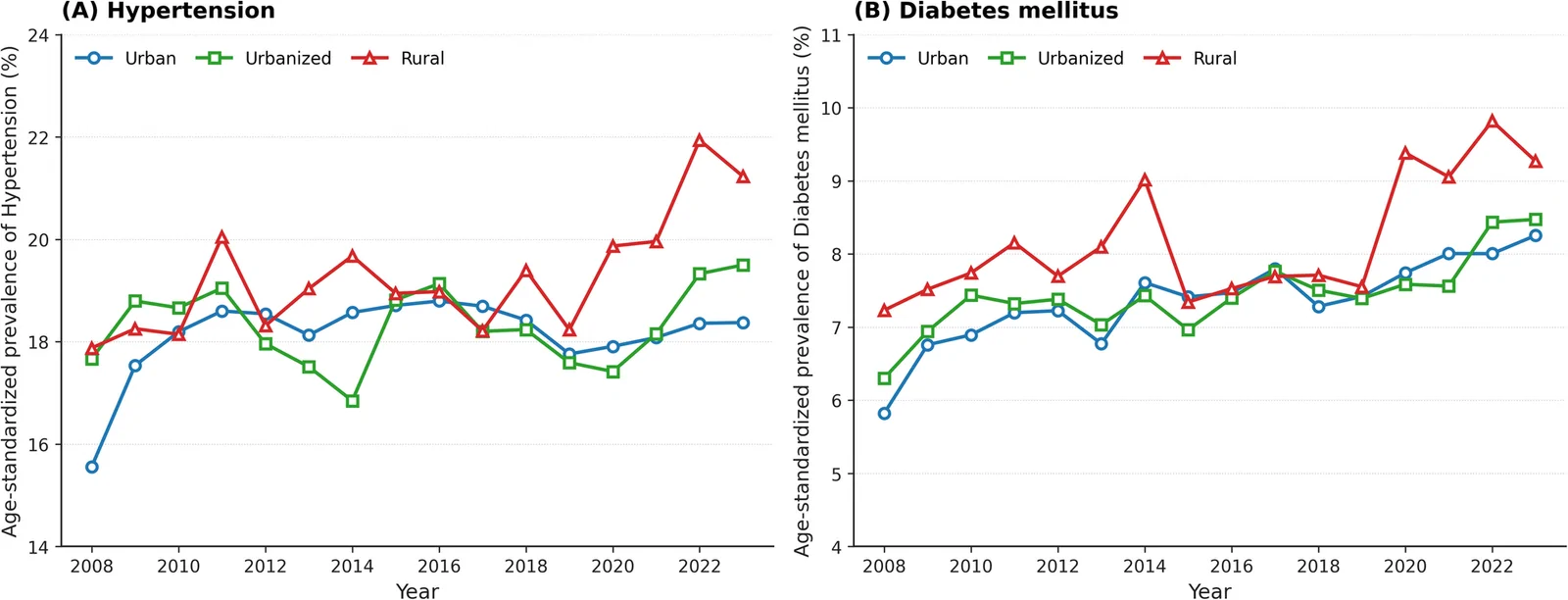
Temporal trends in the age-standardized prevalence of (**A**) hypertension and (**B**) diabetes mellitus by degree of urbanization (DegUrba), Korean Community Health Survey, 2008–2023. Age-standardized prevalence estimates were calculated using the direct standardization method, with the age distribution of the 2020 study population as the standard population. Temporal trends were assessed using general linear model that included year, DegUrba category, and their interaction term. DegUrba, Degree of Urbanization

From 2008 to 2023, age-standardized prevalence of both conditions increased gradually in all DegUrba categories (Fig. 2). Year (linear trend) was a significant predictor for both HTN (*P* = 0.002) and DM (*P* < 0.001) (Table 3). The overall DegUrba group effect was statistically significant for DM (*P* < 0.001) and borderline for HTN (*P* = 0.078), and the year × DegUrba interaction term was borderline for HTN (*P* = 0.077) and non-significant for DM (*P* = 0.694). In pairwise comparisons, the Urban–Urbanized contrast was not statistically significant over the entire 16-year period for either condition (*P* > 0.999), whereas the Rural group differed significantly from both Urban (*P* < 0.001) and Urbanized (HTN *P* = 0.005; DM *P* < 0.001) (Table 3). Age-standardized HTN prevalence in Rural areas rose from 17.9% (2008) to 21.2% (2023), compared with 15.6% to 18.4% in Urban and 17.7% to 19.5% in Urbanized areas. For DM, age-standardized prevalence in Rural areas increased from 7.2% to 9.3%, compared with 5.8% to 8.3% in Urban and 6.3% to 8.5% in Urbanized areas.

**Table 3:** Temporal trends and pairwise group comparisons of age-standardized prevalence by degree of urbanization, Korean Community Health Survey, 2008–2023

Outcome	Effect	Comparison	*P*-value
Hypertension	Year (linear trend)	—	0.002
	DegUrba group	Overall (3 categories)	0.078
	Year × DegUrba interaction	—	0.077
	Pairwise group comparison	Urban vs. Urbanized	> 0.999
		Urban vs. Rural	< 0.001
		Urbanized vs. Rural	0.005
Diabetes mellitus	Year (linear trend)	—	< 0.001
	DegUrba group	Overall (3 categories)	< 0.001
	Year × DegUrba interaction	—	0.694
	Pairwise group comparison	Urban vs. Urbanized	> 0.999
		Urban vs. Rural	< 0.001
		Urbanized vs. Rural	< 0.001

Age-standardized prevalence estimates were calculated using the direct standardization method, with the age distribution of the 2020 study population as the standard population. Temporal trends were assessed using a general linear model including year, DegUrba category, and their interaction term. P-values are reported for the linear effect of year, the overall effect of DegUrba category, the year × DegUrba interaction, and pairwise comparisons between groups. Pairwise P-values were Bonferroni-adjusted

*DegUrba* Degree of Urbanization

## Discussion

### Principal findings

In this cross-sectional analysis of more than 40,000 adults in Gyeonggi-do, four findings emerged. First, the weighted prevalence of HTN and DM was approximately 10% points higher in Rural areas than in Urban or Urbanized areas. Second, Urban and Urbanized areas were statistically indistinguishable in both the single-year 2020 analysis and the 16-year age-standardized trend analysis (Urban vs. Urbanized pairwise *P* > 0.999 for both conditions over 2008–2023). Third, after multivariable adjustment, the rural excess attenuated substantially and was only borderline significant when compared with Urban areas but remained significant when compared with Urbanized areas and became more robust in geographically stable rural municipalities. Fourth, the absolute age-standardized burden in all three categories rose over the past 16 years, with Rural areas maintaining the highest prevalence throughout. Taken together, these results suggest that, within a representative peri-metropolitan province of contemporary South Korea, the cardiometabolic gradient is best characterized not by a smooth urban-to-rural gradient but by a dichotomous “urban/urbanized vs rural” pattern.

### International context: rural cardiometabolic penalty across age, socioeconomic status

The cross-sectional configuration we observed — comparable cardiometabolic prevalence in dense urban and semi-dense urbanized settlements but higher prevalence in rural areas — is consistent with patterns reported from other high-income, aging societies across multiple continents. In the United States, rural residents have substantially higher rates of hypertension and diabetes than urban residents, and the rural–urban gap in cardiometabolic mortality widened over 1999–2018 [14, 15]. In Taiwan, a propensity score–matched analysis of more than 30,000 pairs of newly diagnosed type-2 diabetes patients followed over five years showed that those residing in rural areas had a 15% higher incidence of cardiovascular events, with significantly higher risks of congestive heart failure, stroke, end-stage renal disease, and ischemic heart disease than urban patients [16]. In Japan, a nationwide analysis of 2018 health-checkup data demonstrated that higher area-level deprivation — concentrated in less-urbanized municipalities — was independently associated with a stepwise increase in age-standardized hypertension prevalence and related risk factors [17]. Within Europe, a recent comprehensive assessment of the Nordic countries documented persistent disparities in cardiovascular outcomes and healthcare access between rural and urban districts despite universal welfare systems, particularly affecting elderly residents in sparsely populated areas [18]. Within the Asian region the urban–rural gradient is not unidirectional: large national surveys in mainland China still report higher diabetes and metabolic-risk prevalence in urban than rural residents, although the gap is narrowing as the rural environment changes [10]. The Korean pattern we describe — rural disadvantage in HTN/DM in both the 2020 cross-section and the 16-year temporal trend — therefore aligns most closely with the rural-penalty configuration characteristic of other established high-income, aging societies with universal coverage.

### Urbanization as a health-positive process: why Urban and Urbanized converge

Our most novel observation is the near-identity of Urban and Urbanized cardiometabolic profiles, sustained both in the 2020 cross-section and over the 2008–2023 period. We interpret this as evidence that, in a high-income country with universal coverage, urbanization operates as a discrete “phase change” rather than a smooth gradient: once a settlement crosses the population-density and infrastructure threshold that defines an urban cluster under the DegUrba framework, residents appear to gain access to the same package of cardiometabolic health-promoting features that exists in dense Urban centers. Several mutually reinforcing mechanisms plausibly account for this convergence.

First, healthcare infrastructure scales sharply with population density. In Korea, the density of primary-care clinics, pharmacies, and outreach health workers is markedly higher in urbanized and urban settings than in rural districts, and regional inequalities in healthcare accessibility have been documented at the national level [19]. National analyses of healthy life expectancy by region further show that areas with denser healthcare infrastructure have systematically better population-health outcomes than those without [20].

Second, urbanization is correlated with higher educational attainment and household income, both of which are robustly associated with better awareness, treatment, and control of HTN and DM. National Korean fact-sheet data underline this: among adults with hypertension, awareness, treatment, and control rates are 77%, 74%, and 59% respectively, but these averages mask substantial socioeconomic disparities and are largely driven by patients who can reliably access primary care [21]. For diabetes, integrated achievement of glycemic, blood-pressure, and lipid targets remains modest (around 15%) at the national level but is consistently higher in those with greater education and income [22].

Third, the built environment of urbanized settlements is more supportive of physical activity, social interaction, and incidental exercise than dispersed rural settlements; ambient environmental factors such as air pollution, which independently attenuate the cardiometabolic benefits of physical activity, also vary along the urbanization gradient [23]. Compact street networks, integrated parks, and accessible public transit have been linked to lower cardiovascular morbidity in urban-design reviews. Although these features are typically most developed in dense Urban centers, our data suggest that the threshold at which they begin to deliver measurable health returns is reached in semi-dense Urbanized settlements, helping explain why Urban and Urbanized prevalences converge.

### The Korean context: urbanization as proxy for access to organized chronic-disease management

South Korea’s National Health Insurance Service provides essentially universal financial access to outpatient and pharmaceutical care, but actual utilization of chronic-disease services depends heavily on the local supply of primary care. In rural Gyeonggi-do, the density of clinics, the availability of in-person physician visits, and participation in formal hypertension-management education are all substantially lower than in urban and urbanized districts, and Korean rural residents in regions undergoing population decline are significantly less likely to receive structured hypertension management education and treatment [24]. Telehealth and digital chronic-disease platforms have grown rapidly since the COVID-19 pandemic and were used preferentially by patients in metropolitan and urbanized areas with reliable internet and digital-literacy infrastructure, further entrenching the urbanized advantage in chronic-disease management [25]. Lifestyle data from a Korean national survey also indicate that adverse lifestyle behaviors (poor diet, smoking, insufficient physical activity) are more strongly linked to mortality among rural than urban residents, suggesting that urbanization may also act through the local environment by making behavior-change resources (gyms, nutrition counseling, smoking-cessation services) more readily available where population density supports them [26]. Within Korea, therefore, becoming “urbanized” appears to be defined less by mere population density and more by the acquisition of a comprehensive chronic-disease management system—a process that renders the Urbanized tier functionally equivalent to the dense Urban tier for HTN and DM care.

### What rural areas lack: structural deficits and policy implications

Conversely, the persistent residual rural disadvantage we observed — particularly evident in geographically stable rural municipalities and across the 16-year trend (Urban vs. Rural and Urbanized vs. Rural both *P* < 0.005) — reflects structural deficits that universal insurance does not remedy. Korean rural districts face a self-reinforcing combination of population aging, out-migration of working-age adults, primary-care workforce attrition, and physical distance to specialist services [24]. National health-policy initiatives addressing these structural deficits include the Public Health Doctor program, designated rural Public Health Centers, the Visiting Nurse program for community-dwelling older adults, and, more recently, the Digital Medicine Promotion Act (2023) which provides a legal basis for reimbursement of telehealth and digital therapeutics in medically underserved areas. However, evaluations of these programs consistently identify three remaining gaps: insufficient local primary-care continuity, low rates of structured hypertension-management education in population-declining districts [24], and uneven adoption of digital chronic-disease platforms among the rural elderly [25]. Translating our findings into policy, the most direct corollary is that rural cardiometabolic equity in Korea will require simultaneous strengthening of (i) place-based primary care (e.g., expanded rural-incentive programs for general practitioners, sustained funding of Public Health Centers), (ii) structured outreach for hypertension and diabetes education in population-declining districts, and (iii) age-appropriate digital-health interventions that do not assume baseline digital literacy. From a measurement perspective, the use of the standardized DegUrba framework provides an internationally comparable yardstick by which to evaluate the effect of such interventions over time and across provinces.

### Strengths and limitations

Strengths of this study include the application of the internationally standardized DegUrba framework to a single representative Korean province, the use of the complex KCHS sampling design throughout, the inclusion of a sensitivity analysis restricted to geographically stable municipalities, and the linkage of a single-year multivariable analysis to a 16-year age-standardized temporal trend.

Several limitations should be acknowledged. First, the cross-sectional design of the primary analysis limits our ability to establish definitive causal relationships between the degree of urbanization and the onset of cardiometabolic diseases. Second, hypertension and diabetes were ascertained by self-reported physician diagnosis. Because regular screening is generally less accessible in rural Korea, undiagnosed cases are likely more common there, and the true rural penalty may be larger than that we report. Third, KCHS does not include direct clinical measurements (blood pressure, fasting plasma glucose, HbA1c) or treatment status, and we therefore cannot distinguish between diagnosis, treatment, and control. Fourth, residential mobility was not captured and a “healthy migrant” effect — younger, healthier residents moving to urban centers while older, sicker individuals remain in or relocate to rural areas — cannot be excluded; this is particularly relevant for the interpretation of the compositional adjustment, which may have over-adjusted away part of a place-based effect. Fifth, although we adjusted for major demographic, socioeconomic, and lifestyle factors, residual confounding from occupation, household income, dietary patterns, physical activity, and ambient environmental exposures (e.g., particulate air pollution, which independently affects cardiometabolic risk [23]) cannot be ruled out. Sixth, the analysis was restricted to Gyeonggi-do, the wealthiest and most developed province of Korea, and even its rural strata remain within commuting distance of major urban service hubs. Findings should be applied cautiously to more remote rural areas (e.g., Gangwon province mountainous regions, smaller offshore islands of Jeollanam province and Jeju). Finally, gestational hypertension and gestational diabetes were not separately captured in KCHS and therefore could not be excluded.

### Future directions

Future work should replicate this analysis in geographically and economically distinct Korean provinces — particularly those with a higher proportion of remote rural settlements — and should integrate direct clinical measurements (blood pressure, HbA1c) and treatment/control data from National Health Insurance Service or National Health Screening linkages. Longitudinal designs capturing residential mobility, the timing of urbanization of specific municipalities, and the introduction of place-based interventions would substantially improve causal inference. Comparable application of the DegUrba framework across countries at different stages of the urban transition could clarify whether the urban/urbanized convergence we describe is a generalizable feature of high-income societies with universal coverage.

## Conclusion

Within Gyeonggi-do, the contemporary cardiometabolic burden of hypertension and diabetes is substantially higher in Rural areas than in Urban or Urbanized areas, while Urban and Urbanized areas are statistically indistinguishable both in 2020 and across the 16-year period 2008–2023. Most of the raw rural excess is explained by compositional differences in age, education, marital status, and lifestyle; a small residual rural disadvantage persists, most consistently for hypertension and most robustly in geographically stable rural municipalities. Under universal health coverage, the relevant lever for reducing the rural cardiometabolic penalty is not financial access but place-based strengthening of primary care, structured chronic-disease management, and digital-health adoption in population-declining rural districts. The Degree of Urbanization framework provides an internationally comparable instrument with which to monitor and evaluate such interventions.

## Supplementary Information

Supplementary Material 1

Supplementary Material 2

## Data Availability

The data that support the findings of this study were obtained from the Korean Community Health Survey (KCHS). These data are not publicly available due to restrictions related to data protection and privacy regulations but are available to qualified researchers upon reasonable request and with permission from the respective data providers.
