# Supplementary material for: Degree of urbanization and the prevalence of hypertension and diabetes mellitus in South Korea (Insight from Gyeonggi Province): A cross-sectional analysis with temporal trends, 2008–2023
Source: BMC Public Health. 2026 Jun 19;26:2594. doi: 10.1186/s12889-026-28174-7 (PMC13576108; doi:10.1186/s12889-026-28174-7)
Supplement: Supplementary file 1 [file 12889_2026_28174_MOESM1_ESM.docx]

**Supplementary Table 1. Sensitivity analysis: survey-weighted logistic regression after excluding municipalities whose DegUrba classification changed during the preceding 10-year period**

| **Outcome** | **DegUrba** | **Univariable** | | **Model 1** | | **Model 2** | | **Model 3** | |
| --- | --- | --- | --- | --- | --- | --- | --- | --- | --- |
|  |  | **OR (95% CI)** | **P** | **OR (95% CI)** | **P** | **OR (95% CI)** | **P** | **OR (95% CI)** | **P** |
| **Hypertension** | Urban | *Reference* | | *Reference* | | *Reference* | | *Reference* | |
|  | Urbanized | 1.02 (0.94–1.10) | 0.640 | 0.94 (0.87–1.02) | 0.140 | 0.95 (0.88–1.03) | 0.228 | 0.95 (0.88–1.03) | 0.236 |
|  | Rural | 1.75 (1.60–1.91) | <0.001 | 1.11 (1.01–1.23) | 0.039 | 1.11 (1.01–1.23) | 0.039 | 1.10 (1.00–1.22) | 0.062 |
| **Diabetes mellitus** | Urban | *Reference* | | *Reference* | | *Reference* | | *Reference* | |
|  | Urbanized | 1.03 (0.92–1.15) | 0.612 | 0.96 (0.86–1.08) | 0.527 | 0.98 (0.87–1.10) | 0.711 | 0.98 (0.87–1.10) | 0.741 |
|  | Rural | 1.79 (1.57–2.05) | <0.001 | 1.18 (1.03–1.36) | 0.019 | 1.17 (1.02–1.35) | 0.026 | 1.16 (1.00–1.33) | 0.044 |
| **Hypertension** | Urban | 0.98 (0.91–1.06) | 0.640 | 1.06 (0.98–1.16) | 0.140 | 1.05 (0.97–1.14) | 0.228 | 1.05 (0.97–1.14) | 0.236 |
|  | Urbanized | *Reference* | | *Reference* | | *Reference* | | *Reference* | |
|  | Rural | 1.71 (1.54–1.90) | <0.001 | 1.18 (1.05–1.33) | 0.005 | 1.17 (1.04–1.31) | 0.008 | 1.16 (1.03–1.30) | 0.014 |
| **Diabetes mellitus** | Urban | 0.97 (0.87–1.08) | 0.612 | 1.04 (0.92–1.17) | 0.527 | 1.02 (0.91–1.15) | 0.711 | 1.02 (0.91–1.15) | 0.741 |
|  | Urbanized | *Reference* | | *Reference* | | *Reference* | | *Reference* | |
|  | Rural | 1.74 (1.49–2.03) | <0.001 | 1.23 (1.04–1.45) | 0.014 | 1.20 (1.02–1.41) | 0.030 | 1.18 (1.00–1.39) | 0.049 |

*Abbreviations and model definitions are identical to those of Table 2. The sensitivity analysis was performed to evaluate the robustness of the primary findings by restricting the study population to municipalities with stable DegUrba classification over the recent 10-year period. OR, odds ratio; CI, confidence interval; DegUrba, Degree of Urbanization.*
