# Supplementary material for: Degree of urbanization and the prevalence of hypertension and diabetes mellitus in South Korea (Insight from Gyeonggi Province): A cross-sectional analysis with temporal trends, 2008–2023
Source: BMC Public Health. 2026 Jun 19;26:2594. doi: 10.1186/s12889-026-28174-7 (PMC13576108; doi:10.1186/s12889-026-28174-7)
Supplement: Supplementary file 2 [file 12889_2026_28174_MOESM2_ESM.docx]

**Supplementary Figure 1.** Geographic distribution of the Degree of Urbanization (DegUrba) classification for the 31 municipalities of Gyeonggi-do Province, South Korea, in 2020.

*Municipalities are color-coded by DegUrba category: Urban (red, n=19), Urbanized (purple, n=6), and Rural (gray, n=6). The Seoul Special Metropolitan City and Incheon Metropolitan City, which are administratively separate from Gyeonggi-do, are indicated for reference and were not included in the analysis.*
